# Demolishing the Myth of the Default Human That Is Killing Black Mothers

**DOI:** 10.3389/fpubh.2021.675788

**Published:** 2021-05-24

**Authors:** Stephanie R. M. Bray, Monica R. McLemore

**Affiliations:** ^1^Independent Researcher, Sacremento, CA, United States; ^2^School of Nursing, Department of Family Health Care Nursing, University of California, San Francisco, San Francisco, CA, United States

**Keywords:** Black maternal health, myth, public health, public health praxis, structural racism

## Abstract

It took a white police officer's knee on George Floyd's neck before white people began to reckon with 400 years of slavery and its aftermath, the effects of which Black people have endured for generations. Monuments are being taken down, flags are being redesigned, and institutions that honored those who denied the humanity of Black people are being renamed. Unfortunately for Sandra Bland, Breonna Taylor, Sha-Asia Washington and countless other Black transgender people including those with capacity for pregnancy, there was no justice even prior to the global pandemic of SARS-Cov-2 or coronavirus; namely racism, violence, and the Black Maternal Health crisis that makes it less likely that Black women will survive pregnancy and childbirth. The purpose of this article is to situate the state of Black people with the capacity for pregnancy in the context of these existing crises to illuminate the myths that racism has perpetuated through science, health services provision and policy. The greatest of these is the myth of a default human that can serve as a standard for the rest of the population. This racist ideal underpins education, provision of care, research, policies, and public health praxis. Demolishing the myth starts with acknowledging that Black people are not the architects of their own destruction: the default standard of whiteness is. The article begins with a historical background on how this myth came to be and elucidates the development and perpetuations of the myth of the default human. Next, we present an evidence based scoping review of the literature to summarize current thinking with specific focus on the Black maternal health crisis, we make policy recommendations and retrofits of upstream public health approaches for existing programs toward health equity. We also situate Black maternal health as part of a reproductive justice frame that centers Black women and birthing people's autonomy and agency. In other words, we use the scoping review to end with reimagining public health policy and provide an actionable roadmap to specifically disrupt the myth of the default human and dismantle racism in education, provision of care, research, policies, and public health praxis.

## Introduction

The myth of a default human posits that white people are the natural reference group for all others when designing scientific studies, reporting scientific findings, allocating human, money and time resources, and that the health outcomes of white people in the United States (U.S.) are the best that can be attained. Demolishing this myth starts with acknowledging that Black people are not the architects of their own destruction: the default standard of whiteness is.

The article begins with a historical background on how this myth came to be and essential definitions. The historical background also elucidates the development and perpetuations of the myth of the default human in published scientific and public health literature. Next, we present an evidence based scoping review of the literature to summarize current thinking with specific focus on the Black maternal health crisis. We seek to clarify key concepts and to identify and analyze knowledge gaps. Finally, we make policy recommendations and retrofits of upstream public health approaches for existing programs toward health equity. We also situate Black maternal health as part of a reproductive justice frame that centers Black women and birthing people's autonomy and agency to illuminate how the myth plays out across the reproductive health spectrum. In other words, we use the scoping review to end with reimagining public health policy grounded in reproductive justice and provide an actionable roadmap to specifically disrupt the myth of the default human and dismantle racism in education, provision of care, research, policies, and public health praxis.

This article is unapologetically specific to understanding the experiences of Black people and how scientific racism is manifest in the conduct of clinical and public health research. We begin with some essential definitions to provide readers with clear meanings as they are used in this article. First, where relevant and appropriate we acknowledge that all pregnant capable people do not identify as women, thus we use gender neutral language to foster inclusivity. Second, when citing historical sources and research literature, we retain the language used by the authors. Third, when using the term Black, we make no distinction across diasporas unless specifically noted. In other words, we do not use African Americans to encompass the range of Black people who originate from other geographies, nor do we use Black and African Americans interchangeably. Fourth, we define Black maternal health as the full spectrum of reproductive health experiences that include the perinatal period of pregnancy, labor, birth, and post-partum; when discussing other pregnancy outcomes (i.e., abortion, family planning, miscarriage, surrogacy) we use the more accurate terminology. When discussing people with capacity for pregnancy or pregnant capable people, who are not currently pregnant, we purposively use these terms as opposed to pre-conception. Finally, we include the word mother in our title out of respect to the family members who intentionally use this term in the context of maternal mortality.

### Historical Background

The Black maternal health crisis as we know it today devolved from a system that once deemed Black women the most valuable of all commodities. In 1619, the United States of America was as nascent as its capitalist system, the foundations of which were built on the backs of Africans brought to the Americas as chattel (1–3). After this new nation won its independence, slavery and the growth of capitalism continued hand in hand. Banks, insurance companies, higher education institutions, manufacturing, and health care institutions formed a constellation of enterprises that were created because of and relied on the enslavement of Black people. This began a sustained effort of constructing systems, structures, and policies that inured toward white supremacy and further subjugated Black people.

The U.S. Congress abolished the transatlantic slave trade in 1807 (4). However, interstate slave trade was still legal and under U.S. law, the children of slaves were enslaved by birthright. This made Black women's ability to reproduce paramount. Medical journals and planter records in the British West Indies and the United States reveal growing attention paid by White physicians to enslaved women's reproductive lives (5). Marie Jenkins Schwartz, in her book, *Birthing a Slave: Motherhood and Medicine in the Antebellum South*, noted that:

“Although enslaved midwives and nurses supplied much of the daily plantation health care, slaveowners called upon White physicians for cases such as assisting difficult births with forceps, examining the causes of an enslaved woman's infertility, or investigating cases of infant mortality” (6).

**Figure 1:**
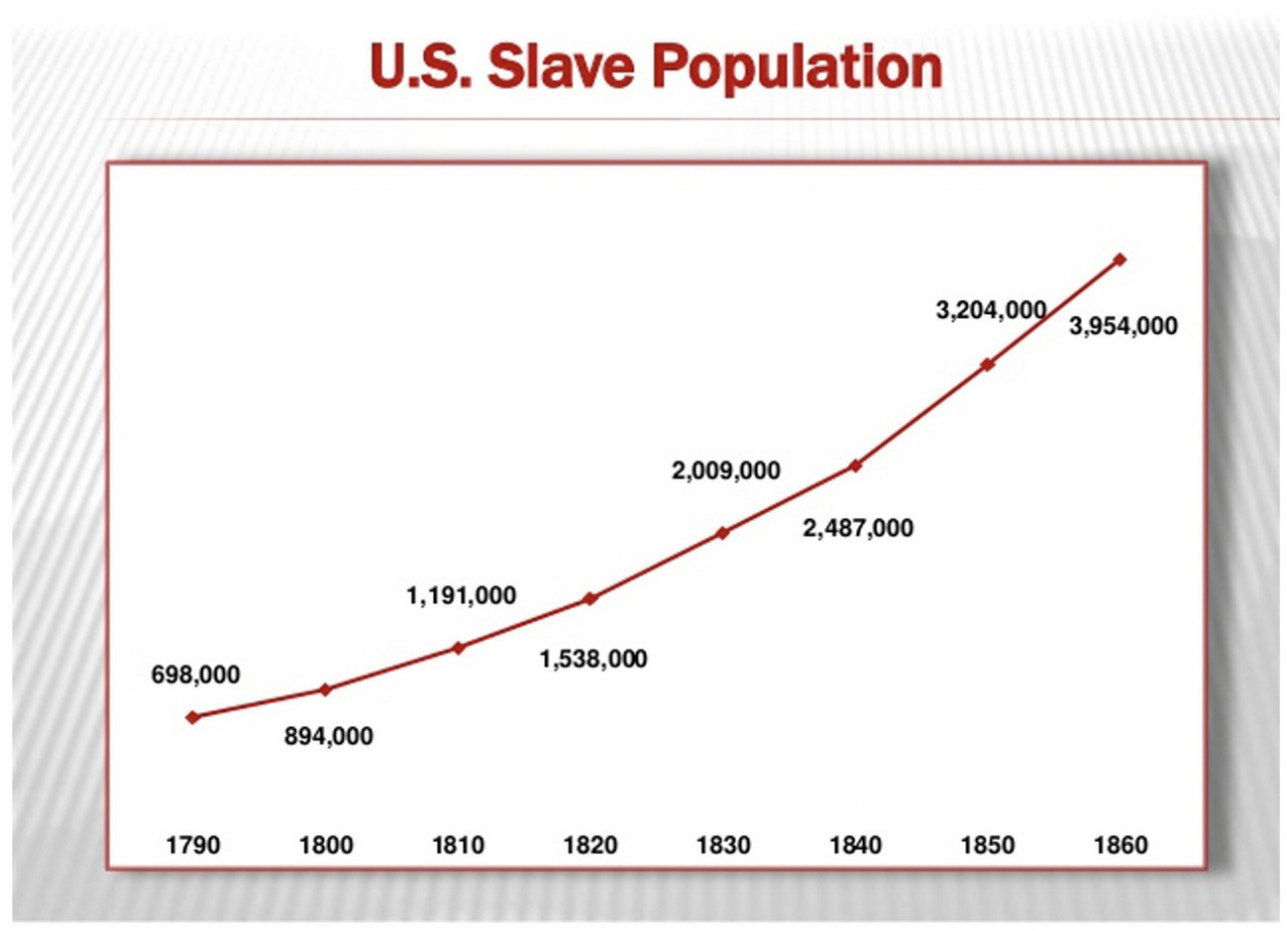
Trajectory of the Slave Population in the United States (U.S.) from 1790 until 1860. This figure used with permission from (copyleft 2007) Chad David Cover. Data derived from “Series A 119–134. Population by Age, Sex, Race, and Nativity: 1790–1970.” U.S. Bureau of the Census, Historical Statistics of the United States, Colonial Times to 1970. Bicentennial Edition, Part 2. Washington, D.C., 1975.

Such surveillance ensured that enslaved Black women continued to reproduce: Between 1807 and 1860, the number of enslaved Black people in the U.S. increased from just over 1 million to over 3.9 million (3). Also see Figure 1.

A lesser-known fact is that while enslaved Black women did not have control over their sexuality or ownership of their bodies, they found ways to manage their fertility (7). By wresting control over their ability to conceive or to bear a child to full term, they declared their whole humanity and agency over their bodies despite a system that would declare otherwise (7). They also defied passing on the birthright of bondage to their children.

Instances of abortion and infanticide are mere mentions in the historical record. According to Deborah Gray White, how enslaved Black women managed their fertility remained a subject discussed only amongst themselves (8). What has been gleaned from further analysis of the Works Progress Administration (WPA) slave narratives is that the ingestion of tinctures made from cotton root, was a form of birth control used to prevent conception. Barrier methods were also used and were dangerous forms of birth control that could result in infection and in some cases, death. These were risks that enslaved Black women believed worth taking. In addition to abortion and infanticide, enslaved Black women practiced abstinence which resulted in further, though inconclusive, surveillance by white doctors who came to believe that those women were simply not able to bear children (7). These examples serve to teach us that the sexual and reproductive health of Black women and their enslaved ancestors is underexamined in historical records. It matters to point these exemplars out to disrupt the myth of the default human: Concerns about reproductive healthcare and injustice did not originate with narratives that are grounded in bra burning and the sexual “revolution” associated with second wave white feminists (9).

The abolition of slavery and the failure of Reconstruction demanded that a hierarchy of humanity be reasserted. While chattel slavery was no longer part of the capitalist system, Jim Crow laws became its proxy, denying freed slaves the rights secured by whites. Laws that mandated segregation based on race gave rise to the development of new systems and structures that excluded Black people. The 1856 ruling in Scott v. Sandford (10) was a bellwether. Central to Scott v. Sanford was Justice Taney's belief that “Black people had no rights that white men were bound to respect,” therefore codifying a racist system of laws established after emancipation (10).

If Scott v. Sanford built the table, Plessy v. Ferguson (11) provided the bounty for *separate but equal*. In 1896, the U.S. Supreme Court ruled against the challenge brought by Homer Plessy that segregation based on race was a violation of the equal protection clause of the 14th Amendment. By ruling in favor of separate but equal accommodations, the Supreme Court essentially relegated Black people to second-class citizenry. The impact of legalized racial segregation and resulting lack of investment in equal housing, education and healthcare for Black people is multi-layered, multi-generational, and profound (12). It is especially broad and deep with regard to access to healthcare, health provision, and the education and training of healthcare professionals.

Limited access to quality public education has had the most profound effect on Black people's health outcomes. After emancipation, the sharecropping system kept Black families in poverty and truncated secondary education. While land grant institutions were founded to educate freedmen, there were many barriers to medical education for Black people, including affordability and access. Racial segregation and structural racism also limited access to residency and specialty fellowship programs. In response to these barriers, a movement to train more Black physicians resulted in the founding of 14 medical schools between the late nineteenth century and early twentieth century. While it has been argued that a few of those schools may have been established as diploma mills that did nothing to advance the cause of medical education for Black people, it is also important to note that most of them were underfunded, lacked adequate facilities, and had limited access to patients (13).

Abraham Flexner in his report (14) commissioned by the Carnegie Foundation in 1910, emphasized the need for improvements in medical education. Flexner visited 155 medical school in 18 months, citing Johns Hopkins Medical School as the standard that medical education should emulate (13). This standard was nearly impossible to meet for medical schools founded to educate Black people. Of the fourteen medical schools founded for that purpose, only two survived: Howard University School of Medicine and Meharry Medical College, which before 1960 had graduated nearly all the Black physicians who received training in the U.S. (15).

Flexner's goal, to improve the quality of medical education, came with a consequence that the healthcare enterprise continues to reckon with. The closure of medical schools founded to educate Black people who were refused admission to majority-white institutions, coupled with the closure of majority institutions that also did not meet the standards that Flexner set, placed medical education further out of reach, especially for Black students. There have been just two Black medical schools founded in the last 100 years: Charles Drew Medical School and Meharry Medical School and <4% of physicians in the U.S. are Black, while Black people make up 13% of the population (13).

Dr. Charles H. Epps, renowned orthopedist, put a finer point on the impact of Flexner's work. In his article, Perspectives From the Historic African American Medical Institutions (16), he wrote that Howard University and Meharry Medical School educated ~85% of all African American physicians whereas the majority medical schools educated 15% for more than half of the twentieth century. Drawing from a 1975 study, Effects of affirmative action in medical schools, Dr. Epps also pointed out that minority physicians, especially African American physicians, tended to provide services in their own communities that are competent and culturally sensitive (17).

For Black people, “The opportunity to train to be a physician is still not where it should be,” Dr. Ed Harley told *MedPage Today*. “More than 100 years later, we are still trying to make up for the deficit” (18).

On the heels of Flexner's report came another set of standards developed without regard for, or understanding of, the historically and culturally relevant experiences of Black women. The Sheppard-Towner Act (19) was birthed after intense lobbying by middle class white women with a progressive agenda for establishing standards to reduce maternal and infant deaths (20). Their work paid off when first, in 1912, the federal government established the Children's Bureau. The Children's Bureau worked with states to track birth and death records under the Sheppard-Towner Maternity and Infancy Act that followed in 1921 (20). Additionally, the Act provided states with federal funding to improve maternal and childcare through education, training and licensing of midwives as well as the establishment of hygiene and other standards. Midwifery was largely concentrated in the rural South where Black (grand) midwives were the majority. Under the scrutiny of Sheppard-Towner, grand midwives were deemed ignorant, dirty, dangerous and prone to superstition (21). The American Medical Association's powerful lobby against the Act due to a fear of government control over the medical profession, was a factor that led to its repeal in 1929 (21). By then the damage was done to Black midwifery: the narrative about the lack of hygiene and cleanliness of Black midwives and Black mothers remained the frame through which Black midwives were trained and through which Black women would continue to be judged as dangerous and not to be trusted.

In her ground-breaking work, *Medical Bondage*: *Race, Gender, and the Origins of American Gynecology*, Dr. Deirdre Cooper-Owens outlines the important connection of these actions on the health of Black people. In a follow-up article for the American Journal of Public Health, she writes:

“Explicit segregation in the realm of health care remained completely intact until the mid-1960s. In 1964, Congress passed the Civil Rights Act, which prohibited federally funded programs and institutions from discriminating on the basis of race. The following year, Congress created the Medicare Program, which made almost all hospitals the recipients of federal funding. As a consequence of their participation in the Medicare Program, almost every hospital in the U.S. was forced to abide by the provisions of the Civil Rights Act of 1964. Despite attempts to prevent racial integration, medical facilities eventually came to treat patients and hire doctors of all races” (5).

While the Civil Rights Act ended de facto segregation, it did not change the fact that Black people were at the bottom of the hierarchy of humanity. As such, they continue to experience seemingly intractable health disparities. In examining the clinical, research and education enterprises within healthcare, these disparities are the result of systematic and intentional adherence to standards built with and toward the exclusion of Black people. Paradoxically, the default standard of whiteness used as the exemplar in the healthcare is also a damnable one: One of the most profound disparities- the high rate of Black women dying during or soon after childbirth- threatens the very ability for Black people to reproduce.

### Scoping Review of the Literature

The process of naming, defining, and documenting a longstanding myth of a default human—that white people are the natural reference group for all others when designing scientific studies, reporting scientific findings, allocating human, money and time resources and that the health outcomes of white people in the U.S. are the best that can be attained—requires rigorous methods. The study of race and racism crosses multiple domains including the arts and humanities, clinical health services provision, healthcare, history, psychology, public health, and sociology. Despite recent attention to health disparities (22), health inequities (23), and anti-racism efforts (24), the boundaries of these domains of knowledge are unknown. To avoid replicating one common mistake of retrofitting new knowledge onto existing science, (i.e., conducting a narrowly defined systematic review), we believe a scoping review of literature is a better approach to synthesizing the myth in context.

The purpose of scoping reviews has been described as the following: (1) To identify the types of available evidence in a given field; (2) To clarify key concepts/definitions in the literature; (3) To examine how research is conducted on a certain topic or field; (4) To identify key characteristics or factors related to a concept; (5) As a precursor to a systematic review; and (6) To identify and analyze knowledge gaps (25), We believe the Black Maternal Health crisis in the U.S. provides a clear exemplar of the manifestation of the myth of the default human. Additionally, given the complexity of how Black maternal health spans social and structural determinants of health, public health, medicine, nursing, health policy, we consider a scoping review as essential to clarify key concepts (i.e., myth of default human and where it manifests) and to identify and analyze knowledge gaps specific to necessary interventions resolve health inequities.

## Materials and Methods

The purpose of the scoping review was two-fold. The first was to clarify key concepts, which requires articles that report data comparing either among Black people and/or between Black people and people of other races to meet inclusion criteria. The second was to achieve our goal of identifying and analyzing knowledge gaps, therefore articles needed to either include interventions in their research methods, report evaluation of interventions, or discuss interventions or mitigation of harm in the section Discussion. Maternal mortality was used as the primary Medical Subject Headings (MeSH) search term including sub-headings of morbidity. The authors conducted the scoping review between December 2020 and February 2021.

Inclusion criteria for the scoping review included articles specific to Humans, published in English and any study conducted and published from any time period with data from people aged 13 to 65 years of age. There were no limitations on the types of research methods used by research teams. Studies reporting individual, community, or neighborhood level data were included. Exclusion criteria included animal studies, studies in languages other than English, systematic reviews of pregnancy outcomes, methodology or methods articles, studies without analyses of African American or Black participants. An additional exclusion criterion was any pregnancy mortality that was not the result of an intended birth, specifically studies describing gestational trophoblastic disease and abortion. Gestational trophoblastic disease is managed as malignancies in oncology and abortion related mortality has been known to be rare, particularly after decriminalization codified in Roe vs. Wade in 1973.

Given the specific focus of racism how and how it operates in the U.S., studies including data from international geographies outside of the U.S. were excluded. However, we acknowledge that the myth of the default human grounded in white supremacy and racism are not exclusive to the U.S. Risk categorization was not considered as an inclusion or exclusion criteria since it is already known that education, income, social and marital status do not impact maternal mortality among Black women—risk is equitably shared regardless of these demographic characteristics.

**Figure 2:**
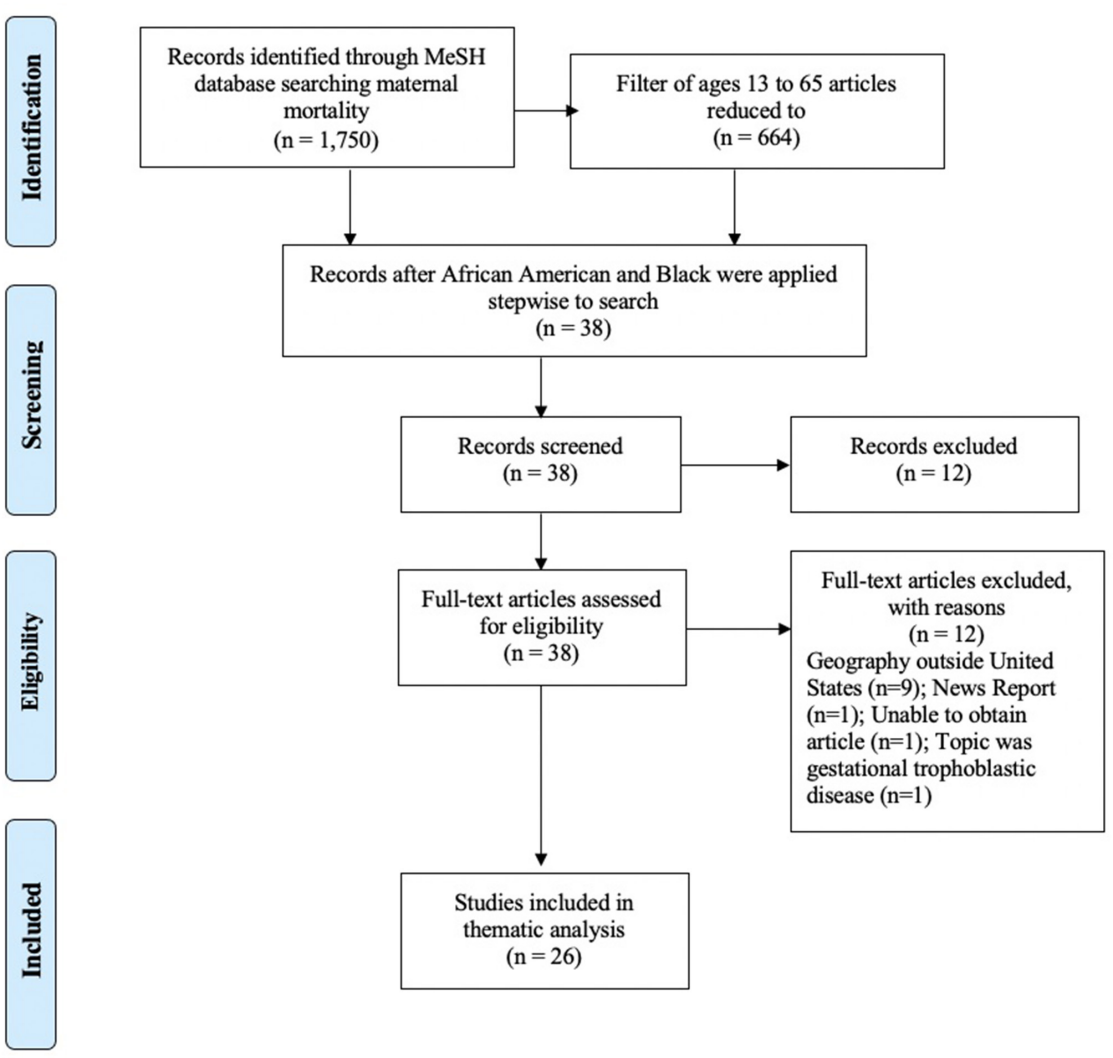
PRISMA for Scoping Review Phase I—Clarify Concepts.

Phase I of the scoping review was conducted to **clarify concepts** and was completed in January 2021 that included articles using the Pubmed MeSH topic *maternal mortality* with no filters and this search resulted in 1,750 articles ranging from 1958 to 2021. When the filters of Human, English language, and ages 13–65 years were set, this reduced the articles to 664. When the word *Black* was applied to this search that yielded 664 articles, the number reduced to 38 articles published between 2000 and 2021. When the term *African American* was applied to the search that yielded 664 articles, the number reduced to 24 articles published between 2000 and 2021. The MeSH search lists (including African American and Black in the search terms) were merged and resulted in 38 articles. All abstracts were retrieved, reviewed and 26 met inclusion criteria for the scoping review. Nine articles were excluded because their study populations were in geographies outside of the U.S, and one article was excluded because it was a news report in a scholarly journal. One abstract could not be obtained. An additional article was excluded because it reported updates in gestational trophoblastic disease—a pregnancy outcome managed differently than birth. The Preferred Reporting Items for Systematic Reviews and Meta-Analyses (PRISMA) for Scoping Review Phase I appears in Figure 2.

**Figure 3:**
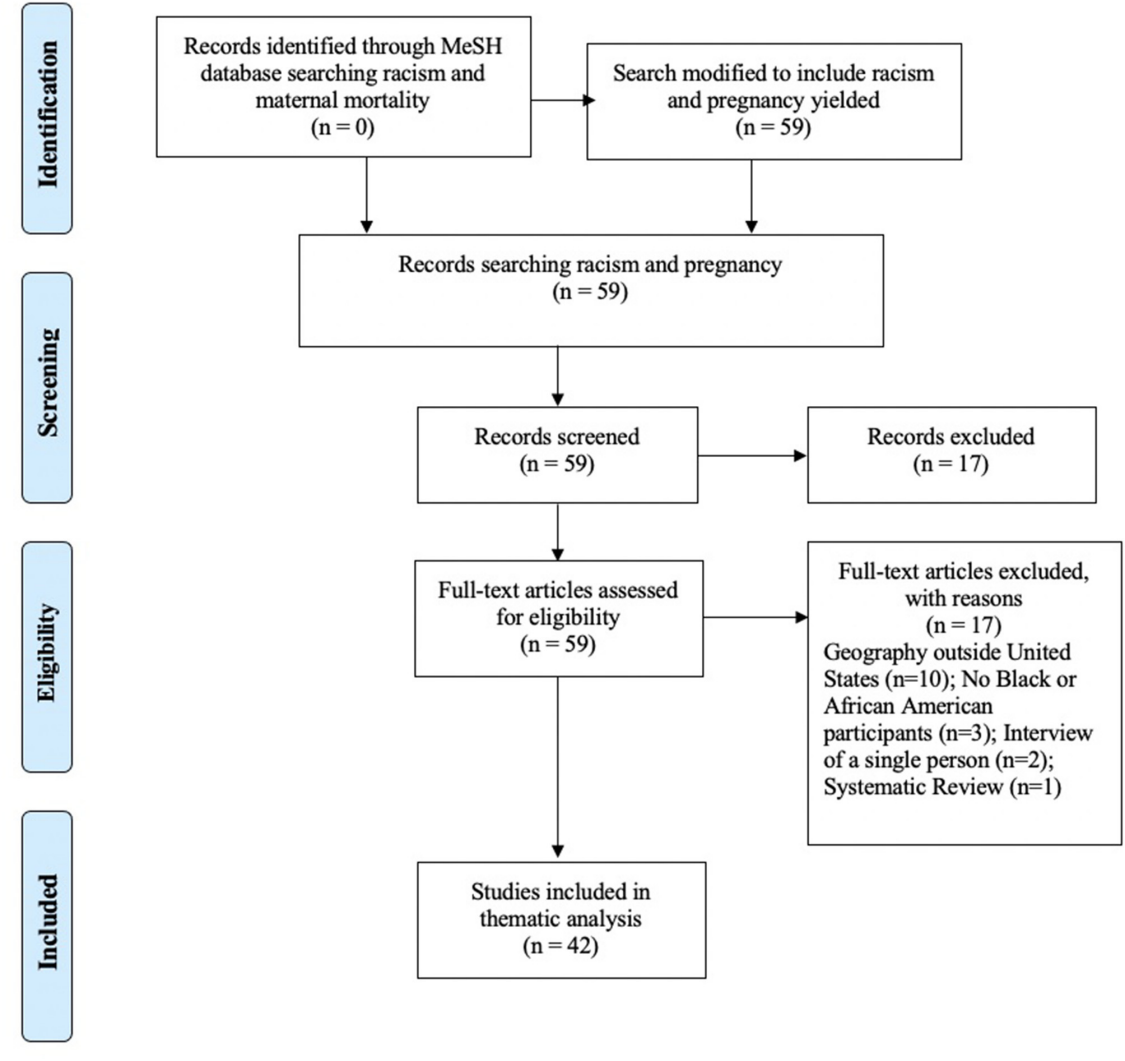
PRISMA for Scoping Review - Phase II - Knowledge Gaps.

Phase II of the scoping review was to **identify and analyze knowledge gaps**. This review, conducted in February 2021, included articles using the Pubmed MeSH topic *racism* with no filters and the addition of *maternal mortality* yielded no results. When *pregnancy* was used instead, this search resulted in 59 articles ranging from 2012 to 2021. No filters were used, and all abstracts were retrieved, reviewed, and 42 met inclusion criteria for the scoping review. Fifteen articles were excluded as the study populations were in geographies outside of the U.S. Three articles did not include Black or African American participants. Two articles were an interview of a single person, and one systematic review of racism and pregnant youth was excluded. Five of the 42 articles included in this scoping review are evidence-based or historical commentaries that use data to make important points specific to interventions and mitigation of harm. The PRISMA for Scoping Review Phase II appears in Figure 3.

In lieu of generating summary statistics, we read each article to synthesize the concepts within and across studies. We used procedures included in thematic analysis methods (26) including familiarizing ourselves with the data; however, instead of generating initial codes, we extracted data for clarifying key concepts specific to establishing the presence/absence of the myth of the default human. By reviewing the full text of the articles that met inclusion criteria, we were able to document themes of each study. We categorized each study based on the type of data that were reported (i.e., individual level data, population level data, and state level data) to determine if these factors impacted the generation of themes. While reviewing themes of each study, we defined, named and categorized themes across studies, allowing us to identify and analyze knowledge gaps.

## Results

A total of 67 published manuscripts combined from Phases I and II were used for this scoping review that includes data that represent both qualitative and quantitative research methods (Supplementary Tables 1–6). Beginning from the foundational work of Guyer et al. (20) which provides the annual summary of vital statistics and trends in the health of Americans during the entire twentieth century, we begin to see the manifestation, development, and perpetuation of the myth of the default human. The a priori categorization of the studies by data level (i.e., individual level data, population level data, and state level data) did not alter the themes, however, for ease of reading grouping them allowed us to determine the elements of the myth of the default human. Themes from the scoping review (Table 1) show there are four elements that are endemic to the maintenance of the myth of the default human that are perpetuated by descriptions of disparities—without testing interventions for mitigation. These elements include (1) Data; (2) Lack of accountability for generation and perpetuation of blame narratives; (3) Life course approaches are under-utilized in study designs; (4) Interventions focus on leveraging existing structures that are inequitable retrofits.

**Table 1:** Thematic analysis of scoping review by phase.

**Phase I (clarifying concepts) theme: data**
• Who is measured and when and where measurement occur
Overreliance on national data sets that are limited.
The outsourcing of data analyses to university-based researchers
Mix of morbidity, mortality and conditions that lead to each
• Attention and focus of research questions
When facility-based analyses are used, few pay attention to staffing, personnel, skill mix or structural factors that impact the facilities
Many analyses are atheoretical
**Phase I (clarifying concepts) theme: lack of accountability for generation and perpetuation of blame narratives**
• No community involvement, engagement or oversight
• Conflation of surveillance statistics and description of disparities
• Dearth of intervention studies; Policy Studies
• Blame-based analytics (i.e., crack cocaine, homicide, gun violence)
**Phase II (knowledge gaps) theme: life course approaches are under-utilized in study designs**
• Establishment of outcomes and exposures
Pregnancy is a condition, and abortion, birth, and miscarriage are outcomes
Selection of control vs. comparison groups (i.e., few within-Black people analyses)
Examinations of maternal death out of context (i.e., life expectancy)
Length of stay analyses
• Family unit analyses
White middle-class lens of analytics
Coupling maternal health and infant outcomes
Ill-defined geographies and rationale for place-based analyses
**Phase II (knowledge gaps) theme: interventions focus on leveraging existing structures that are inequitable retrofits**
• Public health programs such as Doulas, Home Visiting, Midwifery Model of Care, Healthy Start, Women, Infant, and Children Nutrition Program, Family Planning, Nurse Family Partnership, Group Prenatal Care, Social Support
• Medicaid expansion—State focus with inequitable distributions, services, policies

In the Phase I analysis specific to clarifying concepts two themes were identified, data and lack of accountability for generation and perpetuation of blame narratives. Within the data theme were most studies that attempt to define, make sense of, and determine who is measured, when, where, and how. The sub-themes of data are indicative of not having comprehensive national data to understand maternal morbidity and mortality and reflect the limitations of study design when using administrative or publicly available administrative or claims data sets. Specific to lack of accountability, there are several issues that are determined by the fact that Black women and pregnant capable people are not routinely consulted as experts on their own health, nor are their specific research interests reflected in the published literature. Intervention studies are sparce and inadequate because they are focused on leveraging existing structures that are inequitable retrofits (Phase II gap). It is important to note that the bulk of scholarly contributions included in this scoping review have been published within the last 10–20 years (despite no search limits specific to time), reflecting a lack of attention to maternal health more broadly and Black maternal health specifically.

Phase II of the scoping review specific to identifying gaps again focused on data and study design—specifically since the middle-class, white heteronormative gaze is apparent in research questions, the determination of outcomes and exposures without every accounting for the realities of Black life, structural racism, and resilience factors of communities. Additionally, life course approaches were under-utilized in study design. Taken together, one unfortunate conclusion that could be drawn from this scoping review is that the increase in Black maternal death did not draw attention until the data began to indicate an increase in white maternal death. An additional finding from this scoping review suggests the need for a reimagining of education, health services provision, research, and policy specific to the reproductive life courses of Black women and birthing people. Much of the published research treats Blackness as a universal characteristic, with little to no attention to the intersections of class, or income. Despite the fact that it is already known that risk for Black maternal death is equitably shared, interventions have specifically focused on low-income individuals and/or those using publicly funded insurance or services. Given the historical grounding that opens this piece, it should be obvious that the public infrastructure has been inadequate to meet the health needs of Black people. Therefore, to address the findings of the scoping review, that include patients, people, seeking services, as well as clinical treatment and procedures, in context of the historical data presented in the introduction, we propose adopting reproductive justice as a foundational and theoretical frame for intervention research. This path forward should begin essential work to resolve the myth of the default human in the context of maternal morbidity and mortality.

## Discussion

So, what is the way forward? How do we dismantle the myth of the default human and what will it take to build new standards in education, provision of care, research, policies, public health praxis, and workforce development, and ultimately improve Black maternal health outcomes? First, we must acknowledge that the people most impacted by health disparities are best positioned to determine the solutions. This means that Black communities and Black women specifically need to be centered and prioritized in discussions and decisions about Black maternal health. This includes the mapping of assets, and the development and testing of interventions. Best practices and standards in the conduct of research with, for, and by Black mamas have been proposed to improve the quality of research questions that decenter whiteness (27). Next, curricula need to reflect the knowledge production of Black communities and their experiential wisdom. It is disingenuous that patient medical history remains a gold standard of health information gathering, but patient voice is missing from the education and training of the future health care workforce. Third, community-generated big data are essential for research that is conceptualized, operationalized, and actualized with, for, and by Black women. Finally, clinical care needs to be provided by a racially, culturally, and socially concordant workforce. Intentional investment in the healthcare workforce in Black communities that experienced divestment after the Flexner report was released in 1910, is one right place to start.

### Recent Developments and Action Steps

Adopting reproductive justice is an essential component for the design of all clinical health services and educational programs. Specifically, understanding every person has a human right to maintain personal bodily autonomy, have children, not have children, and parent the children we have in safe and sustainable communities (28). If we are to achieve reproductive justice, we can no longer afford to risk the lives of Black women and birthing people based on a mythical standard. Healthcare providers, researchers and scholars, policymakers, and philanthropists–all who have a stake in addressing this crisis–must listen to Black women and birthing people if we are to reduce maternal deaths. We must also remedy the divestments in the social safety net following Reconstruction, the Civil Rights movement, and most of the Reagan era. We cannot allow for a return to complacency if and when we start to see declines in Black maternal morbidity and mortality. History teaches us that any new path forward should center those who are the most marginalized if we are to improve health outcomes for all people with the capacity for pregnancy.

The preservation of Black maternal health is just one aspect of reproductive justice that not only calls for accountability but also requires radical reimagination. This radical reimagination centers Black women, trusts Black women, and invests in Black women. Imagine this: the solution to reducing Black maternal mortality and morbidity already exists within the community that carries the greatest burden. Imagine if Black women defined for themselves the standards by which their agency is measured. Imagine if they harnessed that same self-determination and agency that would have enslaved Black women control whether they would bear a child conceived in rape, to carry and give birth to a much-wanted child to term without sacrificing her life. Imagine if cultural rigor– the operationalization of critical race theory, reproductive justice, research justice, and big data intersected with health services provision, health services research, quality improvement, and health care policy–was used to build those new standards (29). Imagine if the forces that have kept Black people from extinction were better understood and leveraged to improve Black maternal health outcomes.

Recent developments have provided some cause for optimism including a recently announced public-private partnership that was established to address maternal morbidity and mortality from the Department of Health and Human Services, The Office of the Surgeon General, and the March of Dimes (30). Additionally, for the first time since its inception in 2018, a Presidential Proclamation was issued to establish April 11th through April 17th as Black Maternal Health Awareness week (31). Finally, funds were included as part of the American Rescue Plan, for post-partum Medicaid expansion to address preventable maternal mortality—which is known to disproportionally occur in the post-partum period (32).

Additional action steps should include:

- Targeted Investments in Black Students, Educators, Healthcare Providers, and Researchers
- Targeted Investments in the Social Safety Net and Black Communities
- Establishing Community Engagement as a Default Mechanism of Accountability
- Center the Voices, Strategies, and Interventions of Black Birthing People
- Retrofit, Reform, and Reimagine Clinical Health Services Provision, Education, Research, and Policy Development
- Establish Authentic Partnership with Black Women and Femme led Organizations as Leaders of the Work to Reduce Maternal Morbidity and Mortality
- Believe When Interventions are Developed and Implemented to Improve Black Maternal Health that the Health of All Populations Will Improve.

There are limitations and strengths to this work that need to be acknowledged. First, this work is limited to history documented by individuals immersed in and responding to the myth of the default human. We have paid close attention to our citational practices to center the perspectives of Black authors and scholars. Next, articles included in the scoping review were exclusive to those published in English—which misses nuance of Black or African American experiences of people who use speak other languages. Recent work has focused on colorism and the domains of racism and discrimination and this scoping review was unable to be as granular in our analysis—although some of those citations do appear in the review. Finally, we do not proport to have mapped all dimensions of the evidence of the myth of the default human in health services, education, research, and policy related publications. The limited purpose of the scoping review was to clarify concepts and identify gaps to make recommendations about areas where public health interventions could be reimagined.

## Conclusion

In conclusion, we have read or heard countless stories of Black women who have nearly died or have died during or after childbirth. We know them. They are our friends, our neighbors, our co-workers, our family members, and our partners. While we know that higher socioeconomic status is a predictor of health outcomes, this couldn't be further from the truth when it comes to Black maternal health outcomes. How much money or education Black people have, how they construct families, what they wear, their hairstyles, diction, and their very breath are constantly under assault even as Black people aspire to be the “right” kind of people as defined by the default standard of whiteness. It begs the question of whether any default standard is the right exemplar for preventing Black maternal death when we examine the maternal health outcomes in lower income countries where the default standard is not whiteness (29). Too many scholars have been content to describe disparities in reproductive health outcomes specific to Black maternal health, and yet far too few have examined interventions to mitigate the associated drivers of disparities and inequities. Further, the healthcare enterprise continues to dismiss Black maternal agency by perpetuating narratives (e.g., older, sicker, fatter) that place the blame for poor maternal health outcomes on Black mothers (33). Included in that narrative is non-compliance, a by-product of medical mistrust due to racial discrimination (34, 35). None of these approaches appropriately represent the experiences, satisfaction, or quality of Black life or wellness. The inability for research, education, policy or clinical practice to encapsulate or even imagine Black humanity as unique and distinct from the myth of the default standard of white people cannot continue and should be designated unethical. As Katherine McKittrick has outlined in her recent book entitled *Dear Science*, we observe that “the project of making discipline overwhelmingly only gives us two options for the study of Black people—to describe racism and resist racism; these options rarely have any noise or curiosity or questions about Black life interrupting them” (36). We wholeheartedly agree and call upon those in education, provision of care, research, policies, and public health praxis to consider actively dismantling of the myth of the default human—it is not the best that can be attained and new futures remain limited by its continued existence.

## Author Contributions

MM co-wrote this manuscript with SB, principal—Black Women Write. Both authors conceptualized, wrote, and analyzed the data in this manuscript.

## Conflict of Interest

The authors declare that the research was conducted in the absence of any commercial or financial relationships that could be construed as a potential conflict of interest.
