# Supplementary material for: Demolishing the Myth of the Default Human That Is Killing Black Mothers
Source: Front Public Health. 2021 May 24;9:675788. doi: 10.3389/fpubh.2021.675788 (PMC8183820; doi:10.3389/fpubh.2021.675788)
Supplement: Supplementary file 1 [file Table_1.DOCX]

Supplementary Material

Table 1. Phase I List of Individual Level Data Studies Included in Scoping Review.

| **Individual Level Data** | | | | | |
| --- | --- | --- | --- | --- | --- |
| **Study Citation** | **Year Study Conducted** | **Study Method** | **Sample** | **Populations of Study** | **Domains Covered** |
| Brown HL, et. al., 2011 | 1994-2005 | Retrospective Cohort | 12,744 Medicaid Eligible women | Births of all Medicaid eligible women at Duke Medical Center | Near miss categories using WHO criteria* from ICD-9 and DRG data |
| Elkafrawi D., et. al., 2020 | 1986-1996 | Retrospective Cohort | 271 women; singleton birth | African American women with placental abruption | C-section; Crack Cocaine Use; HELLP |
| Frölich MA., et. al., 2014 | 1990-2010 | Case-Control Design | 77 maternal deaths; 154 matched controls | All pregnancy related deaths at UAB Hospital. | Maternal Deaths |
| McFarlane J., et. al., 2002 |  | Case-Control Design  (10 cities) | 197 femicide deaths and 131 attempted femicides; 384 matched controls | Femicide Deaths  Black: 38.1%  White: 30.5%  Hispanic: 24.9%  Other: 6.6% | Maternal Deaths |

*Abbreviations: C-section: Caesarean section; DRG: Diagnosis Related Group; HELLP: Hemolysis, Elevated Liver enzymes and Low Platelet count; ICD-9: International Classification of Disease, 9th revision; UAB: University of Alabama, Birmingham; WHO: World Health Organization*

**Near Miss Categories: Cardiac failure, cardiac arrest, diabetic ketoacidosis, renal failure, respiratory arrest, respiratory failure, stroke, pulmonary edema, uterine rupture, eclampsia, status asthmaticus, shock, disseminated intravascular coagulation, peripartum cardiomyopathy, embolism, and postpartum hemorrhage.*

Table 2. Phase I List of State Level Data Studies Included in Scoping Review.

| **State Level Data** | | | | | |
| --- | --- | --- | --- | --- | --- |
| **Study Citation** | **Year Study Conducted** | **Data Source** | **Sample** | **Populations of Study** | **Domains Covered** |
| Bernet P, et. al., 2020 | 2001-2014 | Single State – Florida | 67 counties | Expenditures in Public Health Programs | Healthy Start; Home Visiting Programs;  WIC |
| Koch AR., et. al., 2019 | 2002-2015 | Single State – Illinois (AHRQ) | 272 confirmed pregnancy related deaths | Black: 47.79% (n=130)  White: 36.76% (n=109)  Hispanic: 12.13% (n=33) | Preventable Maternal Death |
| Koch AR., et. al., 2016 | 2002-2011 | Single State – Illinois (DPH) | 636 maternal deaths (n=82 result of homicide) | Homicide deaths  Black: 52.4%  White: 23.2%  Hispanic: 23.3% | Maternal Death via Homicide |
| Leonard SA., et. al., 2019 | 1997-2014 | Single State – California | 8,252,025  births | Hispanic: 50%  White: 30%  Asian/PI: 12%  Black: 5%  Other: 3%  NI/AN: 0.3% | Severe Maternal Morbidity |
| Mehta PK., et. al., 2020 | 2011-2016 | Single State - Louisiana | 47 confirmed pregnancy related deaths | PAMR data | Preventable death in level 1 to level 4 facilities |
| Momplaisir FM., et. al., 2020 | 2005-2015 | Single State –  Pennsylvania | 905 births among 684 women | Pregnant HIV+ people | Crime; Neighborhood; Poverty |
| Rosenberg D., et. al., 2005 | 1994-1998 | Single State – Illinois | 285 maternal deaths | Hispanic: 11.6%  White: 33.7%  Asian/Other: 3.1%  Black: 51.6% | Maternal morbidity and mortality |

*Abbreviations: AHRQ: Agency for Healthcare Research and Quality-National Inpatient Survey; DPH: Department of Public Health; NI/AN: Native Indigenous/ Alaska Native; PI: Pacific Islander; PAMR: Pregnancy Associated Mortality Review; WIC: Women, Infant, and Children’s Nutrition Program*

Table 3. Phase I List of Population Level Data Studies Included in Scoping Review.

| **Population Level Data** | | | | | |
| --- | --- | --- | --- | --- | --- |
| **Study Citation** | **Year Study Conducted** | **Data Source** | **Sample** | **Populations of Study** | **Domains Covered** |
| Berg CJ., et. al., 2003 | 1991-1997 | National Data (CDC) | 3,201 maternal deaths | PRMR*  All races: 11.5  White: 7.9  Black: 29.6  Other: 11.1 | Maternal Death |
| CDC, 2001 | 1991-1997 | National Data (CDC) | 3,193 maternal deaths | n; PMRM*  All races: 3,193; 11.5  White: 1266; 7.3  Black: 1292; 29.6  Asian/PI: 121; 11.3  Hispanic: 483; 10.3  NI/AN: 31; 12.3 | Maternal Death |
| Chang J., et. al., 2003 | 1991-1999 | National Data (CDC) | 4,200 maternal deaths | Black: 1699 (40.5%)  White: 2293 (54.6%) | Maternal Death |
| Chang J., et. al., 2005 | 1991-1999 | National Data (CDC) | 1993 maternal deaths; 617 (31%) due to homicide | Black: 361 (58.5%)  White: 242 (39.2%) | Maternal Death via Homicide |
| Creanga AA., et. al., 2017 | 2011-2013 | National Data (CDC) | 2009 maternal deaths***** | White: 812 (40.4%)  Black: 761 (37.8%)  Hispanic: 299 (14.8%)  Other: 137 (6.8%) | Maternal Death |
| Creanga AA., et. al., 2015 | 2006-2010 | National Data (CDC) | 3,358 maternal deaths | White: 1352  Black: 1192  Hispanic: 595  Other: 219 | Maternal Death |
| Guyer B., et. al., 2000 | 1632-1998 | National Data (Census) | 100% of birth and death certificates registered in all States and the District of Columbia.** | Foundational Article that includes trajectory of all populations studied in the United States | Maternal Death |
| Miller EC., et. al., 2020 | 1998-2014 | National Data (AHRQ) | 65,286,425 women | White: 42.6% Black: 10.8% Hispanic: 18%  Asian: 3.8%  Other: 4.5% | Hypertension; Stroke |
| Parchem JG., et. al., 2020 | 2014-2017 | National Data (CDC) | 9,205,873 women | White: 55.5% Black: 13.7% Hispanic: 24.3%  Asian: 6.5% | Blood Transfusion; Hysterectomy; ICU admission; Uterine Rupture |
| Metcalfe A., et. al., 2018 | 1993-2012 | National Data (AHRQ) | 12,776,167 women | White: 58%  Black: 15.6% Hispanic: 22.2% Asian: 4.3% | Maternal Death (over time) |
| Mogos MF., et. al., 2020 | 2002-2014 | National Data (AHRQ) | **7,411 inpatient maternal deaths** among an estimated 58,742,179 hospitalizations | White: 42.08% Black: 11.91% Hispanic: 18.78%  Other: 8.55% | Maternal Death |
| Tangel, V., et. al., 2019 | 2007-2014 | Multiple States – CA, FL, KY, MD, NY | 6,872,588 hospitalizations | White: 42.5% Black: 13.9% Hispanic: 28.1%  Other: 12.7% | C-section; Length of Stay; Maternal Death |

*Abbreviations: AHRQ: Agency for Healthcare Research and Quality-National Inpatient Survey; CA: California; C-section: Caesarean section; CDC: Centers for Disease Control and Prevention-National Center for Health Statistics; FL: Florida; ICU: Intensive Care Unit; KY: Kentucky; MD: Maryland; NI/AN: Native Indigenous/ Alaska Native; NY: New York; PRMR: Pregnancy Related Mortality Ratios*

**Pregnancy Related Deaths per 100,000 live births*

***Per Publication Exceptions: “Death data for 1972 are based on a 50% sample of records, as are birth data for 1951 to 1954 and 1956 to1972 and births in selected states during the period 1973 to1984.^4^ Data for years before 1933 are based on estimates developed from birth and death statistics for registration states. More than 99% of births and deaths are currently registered.”*

Table 4. Phase I List of Commentaries, Intervention, and Strategy Studies Included in Scoping Review.

| **Commentaries, Intervention, and Strategy Studies** | | | | |
| --- | --- | --- | --- | --- |
| **Study Citation** | **Study Type** | **Populations of Study** | **Target Audience/**  **Intervention/Strategy** | **Domains Covered** |
| Bingham D., et. al., 2018 | Strategy; Advocacy; Awareness Raising; Education | People in postpartum period | Current Clinical Providers of Care; Family and Care Givers; Policy Makers | Timing of Maternal Death; Signs and Symptoms; Enhancing Postpartum support |
| Gabbe PT., et. al., 2017 | Intervention study from  2011-2014 | Low-income; Medicaid eligible people.  442 births – 6 infant deaths. 339 births – 1 infant deaths | Community-Based Pregnancy Support Groups | Nutrition; Social/Medical Support; Neighborhood/  Place Based Approach |
| Bekemeier B., et. al., 2012 | Population based analysis of NACCHO data from 1993-2005 | Black-white gap analyzed by essential MCH services | 558 counties and multi-county districts | Obstetrical care Family planning  WIC; EPSDT |

*Abbreviations: MCH: Maternal Child Health;* *Medicaid’s Early and Periodic Screening, Diagnosis, and Treatment (EPSDT); NACCHO: National Association of County and City Health Officials; WIC: Women, Infant, and Children’s Nutrition Program*

Table 5. Phase II List of Commentaries Specific to Racism and Pregnancy Included in Scoping Review.

| **Racism and Pregnancy Studies Commentaries** | | | |
| --- | --- | --- | --- |
| **Study Citation** | **Study Design** | **Target Audience/**  **Intervention/Strategy** | **Recommendations** |
| Andaya, E., 2019 | Ethnographic Vignettes | Clinicians; Policy Makers; | Call to attention the structural role of time in public health; Time-pressured providers ; Appointments and wait times; Relationship of time to revenue |
| Frey, CA., et. al., 2014 | Description of a Place-Based Funding Initiative | Clinicians, Policy Makers, Educators, Researchers | **10 Lessons Learned:**  1. An extended planning period helps ground all stakeholders with a common language, build trust, and set the stage for consensus building and collective action  2. It takes time to establish trust and a shared vision  3. Planning enhances the chances of building consensus on strategies. “Planning is doing”  4. The key to sustainability is ensuring an authentic community-driven process  5. The inclusion of all races and all sectors of a community is critical to achieving a collective solutions-driven response to the problem. “Social movements are ignited by the few, but grow in number and strength to impact the masses”  6. The timely transfer of leadership and capacity to local, homegrown persons is important and time consuming  7. Creativity and flexibility are important attributes to achieving large-scale community engagement  8. Acknowledge the presence of racism and be cognizant of how it shapes the fabric of efforts  9. It is important to have a new vocabulary that allows the constructing of messages and narratives that are solution-based and absent of blame shifting. “Health begins where we live, learn, work and play”  10. Descriptive narratives, storytelling and personal testimony, along with one compelling fact are important to making the issue relatable and actionable |
| Gillispie-Bell, V., 2020 | Evidence-Based Commentary | Clinicians; Policy Makers; Academic Administrators | Collective Responsibility; Implicit Bias and Structural Racism; Implementation of IHI Health Equity Principles  Workforce Diversification |
| Perritt, J., 2020 | Evidence-Based Commentary | Clinicians; Policy Makers | Explanation of Criminalization of Pregnant People; Reducing the Role of CPS |
| Scott, KA., et. al., 2019 | Ethics Based Commentary | Clinicians, Policy Makers, Educators, Researchers | Universal preconception care; Strengthening public health programs (i.e., nurse family partnership, group prenatal care, kangaroo care); Adherence to ethical principles outlined by Black Mamas Matter Alliance; Diversification of workforce |
| Silverman, ME., et. al., 2019 | Case-Study and Methods Report | White People; Clinicians; Policy Makers | Reflective Practice; Being versus doing; Therapeutic relationship as facilitator of change; Cultivating self‐awareness; Vulnerability leads to growth; Skill Building |

*Abbreviations: CPS: Child Protective Services; IHI: Institute for Healthcare Improvement*

Table 6. Phase II List of Studies Specific to Racism and Pregnancy Included in Scoping Review.

| **Racism and Pregnancy Studies Commentaries** | | | |
| --- | --- | --- | --- |
| **Study Citation** | **Study Design** | **Study Focus** | **Author Recommendations** |
| Altman, M., et. al., 2019 | Qualitative Interviews | Information Exchange and Healthcare Seeking Experiences | **Change at the individual level**: Provider- and staff-focused education and training respectful care and implicit bias  **Change at the institutional level:** Organizational commitment to upholding a respectful, supportive environment of care, creating flexible schedules for appointments, provision of peer advocates and doulas, and diversification of the provider workforce  **Larger systemic actions:** Re-examination of the organization of care including, insurance and support services, action to dismantle oppressive, structures that enable structural racism and discrimination; Policy actions to better support persons seeking reproductive care; Partnering with communities of color to build new equitable systems of care that address the needs of the community |
| Anthopolos, R., et. al., 2014 | Birth records (2000-2008) for NC | Analysis of racial segregation, isolation, and quality of the built environment | This study suggests that in black segregated neighborhoods, improving the overall quality of the built environment may lead to better birth outcomes among residents |
| Beck AF., et. al., 2019 | Evidence-Based Review | Causal Pathways of how Racism, Segregation, and Inequality impact health | Increased risk; Lower-quality care; and Socioeconomic disadvantages; Removing obstacles, such as discrimination, poverty, and lack of access to quality education, housing, and health care; Develop multiple teams to divide the work. |
| Bower, KM., et. al., 2019 | PRAM Retrospective Cohort Study of 11,582 Black mothers from 2004-2012 from 11 states and NYC | Analysis of Racism and PTB | Patient Centered Care; Addressing Clinician Bias; IHI simulation activities and recommendations |
| Chambers, BD., et. al., 2020 | Place-Based, Repeated Measures Study of 62 Black pregnant and postpartum women from CA | Experiences of racism and discrimination and ICE | Locally monitor and investigate the social determinants of health; Local governments should be held accountable to distribute and track distribution of resources to increase equitable living neighborhoods for Black women |
| Chambers, BD., et. al., 2019 | Place-Based Retrospective Cohort Study | Assess the association between local measures of racial and economic segregation on preterm birth and infant mortality experienced by Black women in CA | Explore how self-reported exposures to racial discrimination moderate the relationship between ICE measures and Black women’s adverse birth outcomes; Analyses of socioeconomic extremes (i.e., educational level and employment status) |
| Chambers, BD., et. al., 2018 | Retrospective Cohort Study 531,170 primiparous women across 33 CA counties | Testing the Association Between Traditional and Novel Indicators of County-Level Structural Racism and Birth Outcomes among Black and White Women | Develop more innovative approaches to: (1) measure structural racism at the county-level and (2) reform public policies to increase integration and access to resources. |
| Christian, LM., et. al., 2013 | Case-control survey and biomarker study of 39 pregnant (19 African American, 20 white) women and 39 non-pregnant matched controls | Analysis of effects of race and pregnancy on stress-induced inflammatory responses | These data represent a promising direction in delineating pathways by which stress may affect pregnancy outcome and fetal development. Stringent inclusion/exclusion requirements increase homogeneity within groups, increasing statistical power – these criteria also exclude women at greatest risk for adverse birth outcomes. |
| Davis, D., 2019 | Qualitative Case-Study Analysis | Analysis of obstetric racism | Adopt reproductive justice approaches to clinical care; Support of Black women led community-based organizations; Cultivating advocacy skills for birthing people. |
| Dillon, B., et. al., 2020 | Cross-sectional Survey of 296 expecting couples from CT | Perceived Discrimination  Naming of double, triple or quadruple jeopardy | “Just in time” feedback; Implicit Bias; Hints at Simulation |
| Dove-Meadows, E., et. al., 2020 | Mixed Methods (surveys, n=38 and interviews, n=7) of Black pregnant women from a single neighborhood | Experiences of racism and discrimination. | Increased MCH nurses and neighborhood assessments;  Individual nurses should scan for personal instances of bias; Referrals to social services |
| Earnshaw, VA., et. al., 2013 | Repeated Measures Survey of 420 women participated (n=62% Latina, n=38% Black) | An evaluation of maternal experiences with discrimination and infant birth weight | Future research should include more specific biological measurements and health complications; Future research might also test whether everyday discrimination, depressive symptoms, pregnancy distress, and pregnancy symptoms measured at different points during pregnancy differentially contribute to birth weight. Future work should continue to compare discrimination experienced at different times in life, both before and during pregnancy, to identify when experiences of discrimination are most detrimental to birth outcomes. Future research should include a larger more diverse sample of pregnant women, and examine other moderators such as nativity status  particularly among Latina women; Future research should continue to explore the nature of discrimination experienced by young, urban women of color. |
| Ertel, KA., et. al., 2012 | Prospective Cohort Study of 2 groups of pregnant Black and African American women (n=877 total) | An analysis of racial discrimination, response to unfair treatment and depressive symptoms | Self-reported racial discrimination was associated with elevated prenatal depressive symptoms during pregnancy among Black women in a cohort that enrolled chiefly low-income women, but this association was not evident in a cohort that enrolled more affluent women. In both cohorts, Black women who talked with others about their experiences of unfair treatment tended to be less likely to experience prenatal EDS |
| Giurgescu, C. et. al., 2017 | Survey of 107 African American pregnant women | Determining if  social support moderates effects of racial discrimination  on psychological wellbeing. | Evaluate the stress experienced by pregnant women due  to their social-structural context such as experiences of  racial discrimination; Provide social support to pregnant women to improve their psychological wellbeing; Establish support groups with a specific focus on issues of  discrimination; Implement stress-reduction interventions;  Develop policy and public health interventions to reduce  racial discrimination or combat its deleterious effects. |
| Giurgescu, C. et. al., 2012 | Descriptive correlational comparative study of African American women (*n*_1_ = 33 with preterm birth; *n*_2_ = 39 with full‐term birth) | An evaluation of neighborhood environment, racial discrimination, psychological distress and PTB. | Data are needed to examine perceived neighborhood environment, which may be more closely related to the stress women experience; Interventions should be developed that support individual women during pregnancy and address neighborhood conditions “‘These interventions would include participation of community partners in decreasing housing vacancy and crime rates.”; Nurses need to advocate for public policies that improve women's living conditions. |
| Hardeman, R., et. al., 2019 | Evidence-Based Call to Action | An application of Beach and colleague's four principles of relationship‐centered care through a critical race lens | Relationships in health care ought to include dimensions of personhood and roles; Affect and emotion are important components of relationships in health care; All health care relationships occur in the context of reciprocal influence; Health care has a moral foundation; New and innovative models of care delivery that center the lived experience of Black people and specifically highlight how racism influences health are urgently needed. |
| Hilmert, C.J., et. al., 2014 | Repeated Measures Survey and Physiological Measures of 39 pregnant African American women | An examination of lifetime exposures of racism and blood pressure fluctuations during pregnancy and fetal growth | Mechanisms that elucidate how racism impacts physiology; For African American women with a history of racism, increases in prenatal diastolic blood pressure may indicate hormonal or immune dysfunction that affects fetal growth |
| Jackson, FM., et. al., 2017 | Survey of 100 mostly low-income African American women | Analysis of Anticipated Negative Police-Youth Encounters and Depressive Symptoms | Support the US Preventive Health Services Task Force released a report recommending universal antenatal and postpartum depression screening; The development and enhancement of interventions that are attendant to the particular cumulative stressors affecting African American women |
| MacDonald, S., et. al., 2017 | National Telephone Based Survey (n=1,341) | Veterans at risk for unintended pregnancy | Veterans Affairs should implement respectful care; Further investigation into women Veterans’ experiences receiving care in the VA system; Further investigation into how intersecting social vulnerabilities (e.g., minority race and sex) may impact health care and outcomes. Research regarding the context in which women Veterans perceive race-based discrimination will be crucial in guiding the continued efforts of VA to meet the needs of the racially diverse population of women it serves |
| Matoba N., et. al., 2019 | Retrospective Cohort (n=33,586) | Analysis of redlining among Black women and preterm birth | Equity in housing law and policy; Inclusion of redlining indices, or other measures of racism, in future public health research are necessary. |
| McLemore, MR. et. al., 2018 | Placed-Based Qualitative Focus Groups | Examination of the healthcare seeking experiences of pregnant, birthing, postnatal Black and women of color | Improved communications, Less reliance of care providers on CPS; Diversification of the healthcare workforce; Support the confidence and competence of birthing people. |
| Mendez, DD., et. al., 2013 | Cohort Study (n=4652) | An analysis of stress (i.e., racism) during pregnancy and residential segregation | Improve our understanding of how external social factors may influence health and stress; Neighborhood social and physical environment may influence stress and health |
| Messer, LC., et. al., 2013 | Retrospective Study of 29 neighborhoods in NC | An evaluation of the relationship between urban built environment and psychosocial health outcomes | Neighborhood indices can be used to evaluate new areas for public health interventions for pregnancy-related behaviors and outcomes; For example, instead of focusing more generally on impoverished neighborhoods, this study has outlined specific neighborhood characteristics that can be focused on for interventions, such as cleaning up disorder or attending to vacant properties; Built environments are modifiable, unlike other risk markers (race, ethnicity) and are therefore important targets for interventions designed to improve public health |
| Padula, AM., et. al., 2020 | Review of Literature | Environmental exposures on perinatal outcomes. Leveraging the NIH ECHO program | Need for more consistent data collection on associations of environmental chemicals and psychosocial stressors and perinatal outcomes |
| Peters, RM., et. al., 2014 | Psychometric Evaluation of the Trust in Physician Scale use with 189 pregnant. African American women | Determination of validity of an existing scale of trust. | The findings of high trust supports a conceptual distinction between interpersonal and social trust; Trust in the provider is interpersonal in nature, built through repeated interactions in which the patient can evaluate the trustworthiness of the provider over time; Social trust is placed in institutions, such as a health care system; Mistrust of social institutions is conceptually distinct from trust in provider but is also affected by previous health care experiences |
| Powell, CA., et. al., 2020 | Place-Based, Repeated Measures Study of 552 dyads from MA from 1999-2002 | Impact of experiences of racial discrimination on infant sleep | Examine mechanisms (Maternal stress, postpartum depression, impact of racism on family unit) |
| Rosenthal, L., et. al., 2015 | Randomized Clinical Trial of Centering Pregnancy© in 7 sites in NY (Repeated Measures Study) | Understanding changes over time specific to discrimination across pregnancy and mental health | Small changes in experiences with discrimination have implications for mental health; Intervening to decrease women’s experiences with discrimination may lead to decreases in their depressive and anxiety symptoms over time. |
| Ruiz, LR., et. al., 2014 | PRAM Retrospective Cohort Study of 2,460 Black and white pregnant people from 2007-2008 from LA | Analysis of health services, provider information and adverse birth outcomes | It is necessary to address racial disparities, cultural competency, and discrimination; Improved measures of racial discrimination during pregnancy and a more thorough understanding of the impact of and pathway from experiences of discrimination to birth outcomes are needed. |
| Salm Ward, TC., et al., 2013 | Focus Group data (n=6 groups; n=29 women; n= 2 individual interviews) | An evaluation of African American women’s perception of prenatal care experiences | Findings highlight the need for a better understanding among providers and systems of care regarding how practices and personal interactions are perceived by the women being served; and How these perceptions may ultimately influence a patient’s use of prenatal care and subsequently, birth outcomes. |
| Slaughter-Acey, JC., et. al., 2019 | Cohort of 1,410 AA women from MI from 2009-2011 | Determination of discrimination and skin tone on entry to PNC | Maternal skin tone, a proxy for colorism, intersects with racial discrimination in the form of racial microaggressions to influence AA women’s use of PNC |
| Slaughter-Acey, JC., et. al., 2019 | Hybrid prospective/retrospective cohort study from MD 872 women from 2000-2004 | Determination of discrimination and racism on SGA infants | Include factors in research  such as racial identity, racial socialization, and race-based traumatic stress |
| Tucker Edmonds, B., et. al., 2017 | Survey of Providers (n=76 OB; n=1 NP) | Case-vignette based assessment of pregnant patients experiencing pain | Provider concern and suspicions are differentially applied based on race. |
| Williams, AD., et. al., 2018 | Retrospective Cohort of stillbirths in Black (n=49,969) and white births (n=71,785) from 2002-2008 | Determine if racial residential segregation is associated with Black-white stillbirth disparities 49,969 black and 71,785 white births. | Decreasing segregation may prevent approximately 900 stillbirths annually among U.S. Black citizens. Reducing structural racism and segregation in particular. |
| Yang, TC., et. al., 2014 | NCHS data (all live births in US except CA) | Examination of smoking, pregnancy, and racial segregation | Public policy aiming to reduce maternal smoking during pregnancy may be more efficient and effective  when strategies specific to racial/ethnic groups are developed and implemented; Specific mechanisms that explain why racial segregation affects maternal smoking during pregnancy behaviors differently for various  racial and ethnic groups; Future research should investigate the mechanisms by which racial integration has deleterious effects on maternal smoking for Asian and Hispanic women and the potential benefits for Black women |

*Abbreviations: AA: African American; CA: California; CT: Connecticut; ECHO: Environmental Influences on Child Health Outcomes; EDS: Elevated Depressive Symptoms; ICE: Index of Concentrations at the Extremes; IHI: Institute of Healthcare Improvement; LA: Louisiana; MA: Massachusetts; MD: Maryland; MI: Michigan; NIH: NC: North Carolina; NCHS: National Center for Health Statistics; National Institutes of Health; NP: Nurse Practitioner; NYC: New York City; PNC: OB: Obstetricians; Prenatal Care; PTB: Preterm Birth; PRAMS:* *Pregnancy Risk Assessment Monitoring System; SGA: Small for Gestational Age; US: United States*
